# Multimorbidity rehabilitation versus disease-specific rehabilitation in people with chronic diseases: a pilot randomized controlled trial

**DOI:** 10.1186/s40814-018-0369-2

**Published:** 2018-11-29

**Authors:** Kathryn Barker, Anne E. Holland, Annemarie L. Lee, Terry Haines, Kathryn Ritchie, Claire Boote, Joanne Saliba, Stephanie Lowe, Fiona Pazsa, Lee Thomas, Monica Turczyniak, Elizabeth H. Skinner

**Affiliations:** 1 Department of Physiotherapy/Community Services, Western Health, 176 Furlong Road, St Albans, Victoria 3021 Australia; 2 La Trobe University, Plenty Rd and Kingsbury Drive, Bundoora, Victoria 3086 Australia; 3 Alfred Health, 55 Commercial Rd, Melbourne, Victoria 3004 Australia; 4 Institute for Breathing and Sleep, Bowen Centre, Austin Health, 145 Studley Road, Heidelberg, Victoria 3084 Australia; 5 Royal Melbourne Hospital, 300 Grattan Street, Parkville, Victoria 3050 Australia; 6 Australian Institute of Musculoskeletal Science, Western Centre for Health Research and Education, Western Health, St Albans, Victoria 3021 Australia; 7 Allied Health Research Unit, Faculty of Medicine Nursing and Health Science, Monash University, Frankston, Victoria 3199 Australia; 8 School of Physiotherapy, Faculty of Medicine Nursing and Health Sciences, The University of Melbourne, Melbourne, Victoria 3000 Australia

**Keywords:** Multimorbidity, Rehabilitation, Exercise, Randomized controlled trial, Cardiac, Pulmonary, Heart failure

## Abstract

**Background:**

Multimorbidity (the co-existence of two or more chronic conditions in an individual) is a growing healthcare burden internationally; however, healthcare and disease management, including rehabilitation, is often delivered in single-disease siloes. The aims of this study were to (1) evaluate the safety and feasibility of multimorbidity rehabilitation compared to a disease-specific rehabilitation program in people with multimorbidity and (2) gather preliminary data regarding clinical outcomes and resource utilization to inform the design of future trials.

**Methods:**

A pilot feasibility randomized controlled trial with concealed allocation, assessor blinding, and intention-to-treat analysis. Seventeen individuals with a chronic disease eligible for disease-specific rehabilitation (pulmonary, cardiac, heart failure rehabilitation) and at least one other chronic condition were recruited. The intervention group attended multimorbidity exercise rehabilitation and the control group attended disease-specific exercise rehabilitation. Participants attended twice-weekly exercise training and weekly education for 8 weeks. Feasibility measures included numbers screened, recruited, and completed. Other outcome measures were change in functional exercise capacity (6-minute walk test (6MWT)), health-related quality of life (HRQoL), activities of daily living (ADL), and resource utilization.

**Results:**

Sixty-one people were screened to recruit seventeen participants (nine intervention, eight control); one withdrew prior to rehabilitation. Participants were mostly male (63%) with a mean (SD) age of 69 (9) years and body mass index of 29 (6). The intervention group attended a mean (SD) of 12 (6) sessions, and the control group attended 11 (4) sessions. One participant (6%) withdrew after commencing; two (12%) were lost to follow-up. The intervention group 6MWT distance increased by mean (SD) of 22 (45) meters (95% confidence interval − 16 to 60) compared to 22 (57) meters (95% confidence interval − 69 to 114) (control).

**Conclusions:**

It was feasible to recruit people with multimorbidity to a randomized controlled trial of rehabilitation. A large RCT with the power to make significant conclusions about the impact on the primary and secondary outcomes is now required.

**Trial registration:**

The trial was registered with the Australian and New Zealand Clinical Trials Registry available at http://www.anzctr.org.au ACTRN12614001186640. Registered 12/11/2014.

**Electronic supplementary material:**

The online version of this article (10.1186/s40814-018-0369-2) contains supplementary material, which is available to authorized users.

## Background

Multimorbidity (the co-existence of two or more chronic conditions in an individual) [1] is a growing healthcare burden internationally [2–4]. Two thirds of adults over 60 years have multimorbidity [5], the severity increasing with age [6]. With an estimation that 25% of our population will be over 65 years of age by 2015 [7], the prevalence of multimorbidity will rise significantly. This is of importance to the healthcare system with multimorbidity associated with increased premature mortality [8, 9], poorer functional status [10], and reduced health-related quality of life (HRQoL) [11].

Worldwide healthcare delivery focuses on single diseases [1, 12, 13]. This challenges those caring for patients with chronic disease, as multimorbidity has direct management implications. Single-disease clinical guidelines are not developed in a multimorbidity context nor consider multimorbidity [14–16]. People with multimorbidity are managed with multiple single-disease guidelines. However, recent multimorbidity guidelines suggest that single-disease care may not be appropriate for people with multimorbidity, due to the potential interactions between diseases and drugs as well as total treatment burden [17].

Rehabilitation is integral to chronic disease management but is frequently structured in single-disease siloes such as cardiac and pulmonary rehabilitation. Meta-analyses demonstrated improvements in exercise capacity, symptoms, HRQoL, and reduced hospitalization in people with chronic disease [18–21]. However, patients attending the disease-specific rehabilitation programs are increasingly complex with more co-existing health conditions. In the United Kingdom (UK), 46% of patients in cardiac rehabilitation have comorbidities [22]. While patients with multimorbidity are included in cardiac, heart failure (HF), and pulmonary rehabilitation, their clinical outcomes are less optimal compared to people with single diseases [23–25].

An alternative option is multimorbidity rehabilitation, with inclusive criteria that do not limit participation due to disease type. Understanding whether the provision of multimorbidity rehabilitation for this population is at least equivalent in health outcomes to disease-specific rehabilitation has considerable implications. People with multimorbidity may benefit from a modified structure which accommodates all conditions and which influences their benefit from rehabilitation. Multimorbidity rehabilitation also addresses recommendations that a care model should aim to improve HRQoL by reducing treatment burden, adverse events, and unplanned care [17].

The aims of this study were to (1) evaluate the safety and feasibility of a multimorbidity rehabilitation compared to a disease-specific rehabilitation program in people with multimorbidity and (2) to gather preliminary data regarding proposed outcomes for the main trial which were change in functional exercise capacity, activities of daily living (ADL), HRQoL, and resource utilization.

## Methods

### Study overview, design, and setting

This trial was a pilot feasibility single-blind parallel randomized controlled trial (RCT), conducted at Sunshine Hospital, Victoria, Australia. Participants were recruited from November 2014 to February 2015 and sourced from referrals to pulmonary, cardiac, and HF rehabilitation programs, inpatient medical, respiratory, and cardiology wards, and the community-based rehabilitation service at Western Health. Ethical approval was obtained from the Melbourne Health Human Research Ethics Committee and La Trobe University. The trial was registered with Australian New Zealand Clinical Trials Registry (ACTRN 12614001186640) and reported according to CONSORT guidelines [26].

### Eligibility criteria

The inclusion criteria were adults (aged > 18) with a physician diagnosis of a single disease for which usual care rehabilitation was indicated (i.e., Chronic Obstructive Pulmonary Disease (COPD), bronchiectasis [27], HF, coronary artery disease or ischemic heart disease [28]), and at least one other chronic condition, such as diabetes, hypertension, and cancer [1]. Exclusion criteria were an inability to walk 50 m, severe cognitive impairment, unstable cardiovascular disease or diabetes, and confirmed pre-existing participation in a structured exercise program. A detailed list of eligible chronic conditions and exclusion criteria are included in Additional file 1.

### Randomization

Eligible participants were randomized in a 1:1 allocation. The allocation sequence was generated using computer-generated random numbers and group allocation was placed into sealed opaque envelopes by an independent investigator not involved in intervention delivery or outcome measurement. Randomization occurred after the signing of the consent form and completion of the baseline data collection.

### Interventions

Participants were randomized to either usual care disease-specific rehabilitation program (pulmonary, cardiac, or HF rehabilitation) (control) or a multimorbidity rehabilitation program (intervention). Both rehabilitation programs were 8 weeks duration and comprised exercise (1 h, twice-weekly) and education (1 h, once weekly) performed in an outpatient setting. The interventions were delivered by health professionals experienced in the delivery of disease-specific rehabilitation programs. A face to face instruction session was conducted prior to the commencement of the intervention period to ensure consistent delivery of exercise.

#### Exercise

Exercise prescription and delivery was equivalent in the disease-specific and multimorbidity rehabilitation programs. Clinicians were encouraged to individualize the exercise program to accommodate participants’ chronic conditions. For example, a second walking session was included to replace cycling if the participant was unable to use a stationary bike due to back pain. The program consisted of aerobic (walking and cycling) and strengthening (upper and lower limb) exercises (see Additional file 1 for exercise details).

#### Education

Education for the disease-specific and multimorbidity rehabilitation programs was delivered by multidisciplinary team members using a didactic approach with handouts provided. (Table 1). The disease-specific topics were consistent with the current recommendations [28, 29]. The multimorbidity rehabilitation program education sessions aimed to enhance skills in general disease self-management and focused on common risk factor modification for chronic diseases [30]. Participants were directed towards finding relevant information and resources in disease management. The “managing morbidity” session aimed to teach participants to recognize when their disease symptoms changed and to develop their relationship with their general practitioner (GP) to manage these changes or exacerbations, rather than addressing disease-specific action plans. The multidisciplinary team presenting the education did not cover any disease-specific topics, but addressed specific questions that arose during the sessions. A diabetes education session was included in the multimorbidity education due to the high prevalence of diabetes in the study population. The pharmacy education session did not present the common medication classes or device techniques for specific diseases, as is usual in disease-specific rehabilitation, but focused on awareness of community services commonly available through local pharmacies to assist people with managing polypharmacy, such as home medication review and medication distribution packs. Some of the presentations were developed collaboratively by the study team (i.e., managing multimorbidity) and others by individual disciplines (i.e., dietetics and psychology). Several of the presentations were adapted from existing disease-specific presentations. Goal setting was a core component of the sessions, without the use of specific behavior change techniques.

**Table 1:** Education sessions

	Multimorbidity	Usual disease-specific		
		Pulmonary rehab	Cardiac rehab	Heart failure rehab
1	Nursing What is multimorbidity? Managing multimorbidity—risk factors and setting goals Finding useful resources	Speech pathology Managing shortness of breath and eating and talking Finding useful resources	Social work Services and supports Social supports Finding useful resources	Social work Services and supports Social supports Finding useful resources
2	Nursing Communication with health care professionals, family, and friends Smoking cessation Blood pressure and cholesterol—how to manage.	Nursing What is respiratory disease? Managing your disease (action plans) Smoking cessation	Nursing What is heart disease? Managing your disease (action plans) Risk factor modification	Nursing What is CHF? Managing your disease (action plans) Risk factor modification
3	Physiotherapy Why is exercise important? Types of exercise and how much to do Precautions and warnings for exercise	Physiotherapy Why is exercise important? Types of exercise and how much to do Precautions and warnings for exercise	Physiotherapy Why is exercise important? Types of exercise and how much to do Precautions and warnings for exercise	Physiotherapy Why is exercise important? Types of exercise and how much to do Precautions and warnings for exercise
4	Dietetics Healthy Eating Weight management Finding useful resources	Dietetics Healthy Eating Weight management Finding useful resources	Dietetics Healthy Eating Weight management Finding useful resources	Dietetics Healthy Eating Weight management Finding useful resources
5	Diabetes Educator What is diabetes? Managing blood sugar levels Signs and symptoms of low/high blood sugar levels	Continence What is incontinence? Managing incontinence Finding useful resources	Diabetes educator What is diabetes? Managing blood sugar levels Signs and symptoms of low/high blood sugar levels	Diabetes educator What is diabetes? Managing blood sugar levels Signs and symptoms of low/high blood sugar levels
6	Pharmacy General medicine advice Why am I taking so many medications? Home medicine review	Pharmacy Inhalers and medications Why am I taking so many medications? Home medicine review	Pharmacy Classes of medications Why am I taking so many medications? Home medicine review	Pharmacy Classes of medications. Why am I taking so many medications? Home medicine review
7	Occupational therapy Performing activities of daily living Energy conservation Relaxation and stress management	Occupational therapy Performing activities of daily living Energy conservation Relaxation and stress management	Occupational therapy Performing activities of daily living Energy conservation Relaxation and stress management	Occupational therapy Performing activities of daily living Energy conservation Relaxation and stress management
8	Psychology Anger/shock/numbness/denial/disbelief Acceptance and building problem-solving skills Action towards achieving a modified healthy lifestyle	Psychology Anger/shock/numbness/denial/disbelief Acceptance and building problem-solving skills Action towards achieving a modified healthy lifestyle	Psychology Anger/shock/numbness/denial/disbelief Acceptance and building problem-solving skills Action towards achieving a modified healthy lifestyle	Psychology Anger/shock/numbness/denial/disbelief. Acceptance and building problem-solving skills Action towards achiving a modified healthy lifestyle.

### Outcome measures

Initial and discharge assessments were conducted at baseline and following rehabilitation completion by blinded assessors. The blinded assessors were provided with a face to face instruction session and manuals for performing the measures prior to the commencement of the data collection.

Baseline demographics, medical history, and multimorbidity measures [11] were collected. The use of multiple multimorbidity measures was to determine which measures would be most suitable for a larger scale trial for ease of use and information obtained. These included the Cumulative Illness Rating Scale for Geriatrics (CIRS(G)) [31], the Functional Comorbidity Index (FCI) [32], the Multimorbidity Illness Perception Scale (MULTIPleS) [33], and the Duke Severity of Illness Checklist (DUSOI) [34]. The detailed information regarding these measures is in the Additional file 1.

#### Feasibility and process outcomes

The program feasibility was measured by numbers screened to achieve target sample size, the number of those who agreed to participate; and the number who completed the intervention. Program completion was defined a priori as attendance at 12 out of 16 sessions for pulmonary rehabilitation [35]; similar cutoffs for cardiac, HF, and multimorbidity rehabilitation were applied for consistency.

### Patient-centered outcomes

#### Functional exercise capacity

The primary outcome proposed for the main trial was change in functional exercise capacity, measured by the 6-min walk test (6MWT). The 6MWT has demonstrated validity and reliability in patients with chronic respiratory disease [29], HF [36], and in patients with cardiac disease and multi-morbidities [37]. The 6MWT was administered according to guidelines, with two tests conducted, with the longest distance recorded [38]. Supplemental oxygen was delivered during the 6MWT for any participant who was normally prescribed with domiciliary exertional oxygen with the same flow rate used at each assessment.

Secondary outcomes proposed for the main trial included ADL and HRQoL questionnaires and resource utilization.

#### Activities of daily living

Functional ADL were measured using the Katz Index of Independence in Activities of Daily Living (Katz ADL index). The Katz ADL index is used in older people to measure function [39] and has been used in people with chronic diseases [40].

#### Health-related quality of life

HRQoL was measured with all participants using two generic instruments, the Assessment of Quality of Life (AQoL-4D) [41, 42] and EuroQol-5D-5 L (EQ-5D-5 L) [43, 44]. The AQoL-4D and EQ-5D-5 L are valid and reliable instruments, with moderate levels of responsiveness and sensitivity in a wide range of health conditions [41, 44]. The EQ-5D-5 L may be considered as a second potential primary outcome. Disease-specific HRQoL measures were the Minnesota Living with Heart Failure Questionnaire (MLHF) for participants with a primary diagnosis of HF and St. George’s Respiratory Questionnaire (SGRQ) for participants with a primary diagnosis of respiratory disease. The SGRQ and MLHF are reliable and valid instruments that are sensitive and responsive to change after pulmonary rehabilitation or exercise training for people with HF [29, 45].

#### Resource utilization

Resource utilization was measured by collecting data on emergency department (ED) presentations, hospital admissions, GP presentations during the trial period, and any health event necessitating hospital admission during the intervention. Participants also maintained a daily diary recording all medical consultations with their GP or consultant physician and hospital admissions. Diary information was verified by participant interview at the post-intervention assessment. Hospital admissions and length of stay were verified using Western Health patient medical records.

### Statistical methods

#### Sample size

As this was a pilot trial, no sample size calculation was undertaken [46]. A sample of sixteen participants was recruited due to the resources available and the timeframe to complete the intervention.

#### Statistical analysis

Feasibility was described in numbers and percentages. Continuous variables were reported as mean and standard deviation (SD), or median and interquartile range [IQR] depending on data distribution. Continuous variables were analyzed using a paired or independent t-tests for normally distributed data and Chi-square or Mann-Whitney U test for non-normally distributed data. All patient-centered outcomes were presented with a 95% confidence interval (CI). Data analysis was by intention-to-treat. The study was not powered to detect differences in patient-centered outcomes and therefore, the results of hypothesis testing should be interpreted with caution. Data were analyzed through the Statistical Package for the Social Sciences (SPSS) Windows Version 23.0.

## Results

**Fig. 1:**
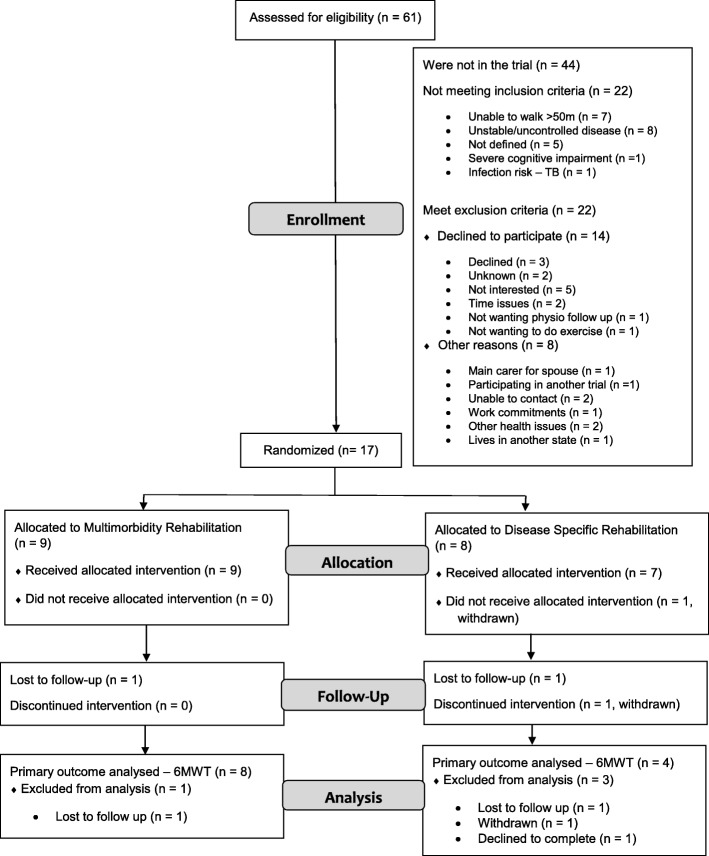
CONSORT flow diagram of patient flow through the study

Sixty-one people were screened to recruit 17 participants (Fig. 1). The original aim was to recruit 16 participants; however, one participant withdrew prior to intervention and therefore an additional participant was recruited. Of the 44 not in the trial, 22 did not meet the inclusion criteria. Of these, eight had unstable or uncontrolled disease. Seven were excluded as they were not able to walk more than 50 m. Of the 22 who met the criteria but were not recruited, 14 declined to participate with the most common reason “not being interested” in five. Other reasons for declining are in the Additional file 1. Of the 17 participants recruited and randomized, nine were randomized to the Multimorbidity Rehabilitation Group (MMRG) and eight to the Disease-Specific Rehabilitation group (DSRG). All nine of the MMRG received the intervention and seven in the DSRG received the intervention, with losses to follow up detailed in Fig. 1. Two participants from the MMRG (none from the DSRG) who did not complete the rehabilitation program were included in the analysis as they completed all post-intervention outcome measures.

Participant demographic characteristics are summarized in Table 2. A total of 63% were male, with a mean (SD) age of 69 (9) and body mass index of 29 (6). The MMRG had a higher baseline 6-min walk distance (6MWD) of 446 (102) meters (m) compared to the DSRG with 335 (141) m.

**Table 2:** Participant characteristics

	Multimorbidity Rehabilitation Group (MMRG) (*n* = 9)	Disease-Specific Rehabilitation Group (DSRG) (*n* = 7)
Age (in years), mean (SD)	68 (10)	71 (8)
Male, *n* (%)	7 (78%)	3 (43%)
BMI, mean (SD)	27 (6)	32 (5)
Main diagnosis, *n* (%)		
- Acute myocardial infarction	2 (22%)	1 (14%)
- Percutaneous coronary intervention—stent	0 (0%)	3 (43%)
- Coronary artery bypass graft surgery	2 (22%)	0 (0%)
- Mitral valve replacement/repair	1 (11%)	0 (0%)
- Chronic obstructive pulmonary disease	3 (34%)	2 (29%)
- Asthma	0 (0%)	1 (14%)
- Congestive heart failure	1 (11%)	0 (0%)
Disease-Specific Program Originally Referred to, *n* (%)		
- Cardiac	5 (56%)	4 (57%)
- Heart failure	1 (11%)	0 (0%)
- Pulmonary	3 (33%)	3 (43%)
Smoking status, *n* (%)		
- Current	1 (12%)	1 (14%)
- Ex-smoker	4 (44%)	4 (57%)
- Never	4 (44%)	2 (29%)
Baseline 6MWD, mean (SD)	446 (102)	335 (141)
Other comorbidities, *n* (%)		
- Coronary heart disease	8 (89%)	4 (57%)
- Hypertension	8 (89%)	5 (71%)
- Diabetes	4 (44%)	4 (57%)
Number of comorbidities, mean (SD)	4 (1)	4 (1)
Functional Comorbidity Index, mean (SD)	4 (2)	8 (1)
Multimorbidity Illness Perception Scale, mean (SD)		
- Treatment burden	4 (3)	5 (4)
- Prioritization	6 (4)	9 (2)
- Causal relationships	3 (3)	3 (3)
- Activity restriction	4 (3)	4 (2)
- Emotional representations	9 (12)	16 (9)
- Summary scale	26 (20)	37 (16)
Cumulative Illness Rating Scale for Geriatrics, mean (SD)		
- Total number of categories endorsed	4 (2)	5 (1)
- Total score	6 (3)	9 (2)
- Severity index	2 (0)	2 (0)
- Number of categories at level 3 severity	0 (1)	1 (1)
- Number of categories at level 4 severity	0 (0)	0 (0)

*SD* standard deviation, *n* number, *BMI* body mass index, *6MWD* 6-min walk distance

The DSRG had a higher FCI compared to the MMRG, indicating lower physical function [32]. The higher summary score for the MULTIPleS in the DSRG indicated worse perception of their multiple diseases [33]. For the CIRS(G), the higher total score for the DSRG compared to the MMRG suggests greater medical burden in this group [31]. However, the severity index score was the same for both groups with a small difference in the total number of categories endorsed (Table 2), indicating a higher level of severity of disease or more chronic problems in the DSRG. The DUSOI data was not reported due to issues encountered in tool use. All assessors found the tool difficult to use and several assessors administered the tool incorrectly, by asking participants to select categories rather than the clinician deciding.

Fifty percent of the participants required individual adjustments to their exercise program to accommodate their multimorbidity. The causes of the adjustments were pain located in the knee (*n* = 6), hip (*n* = 1), and back (*n* = 3), and balance issues in two participants. Of the eight participants requiring modification to the exercise prescription, three had multiple causes and five had a single cause. Both participants with balance issues required adjustment of the step exercise (a lower limb strengthening exercise), where they were unable to hold weights in their hands and safely complete the step action. Therefore, one participant held a weight in one hand and placed the other on a rail and one placed both their hands on rails and did not hold any weights. The pain (hip, knee, and back) issues affected participants’ ability to perform the following exercises: squats, steps, sit to stand (lower limb strengthening exercises), cycling (aerobic exercise), and upper limb weights (upper limb strengthening exercise). The adjustments included repetition of an alternative exercise component (e.g., walking, sit to stand, and squats), not performing that exercise, performing the upper limb exercises in a seated position, or increasing the sets and repetitions of another exercise component.

### Post-intervention

Overall, 63% of the participants completed the rehabilitation programs (67% in MMRG compared to 57% in DSRG). The MMRG attended a mean number of 12 (6) sessions and the DSRG attended 11 (4) sessions. No adverse events related to the interventions or testing were recorded.

**Fig. 2:**
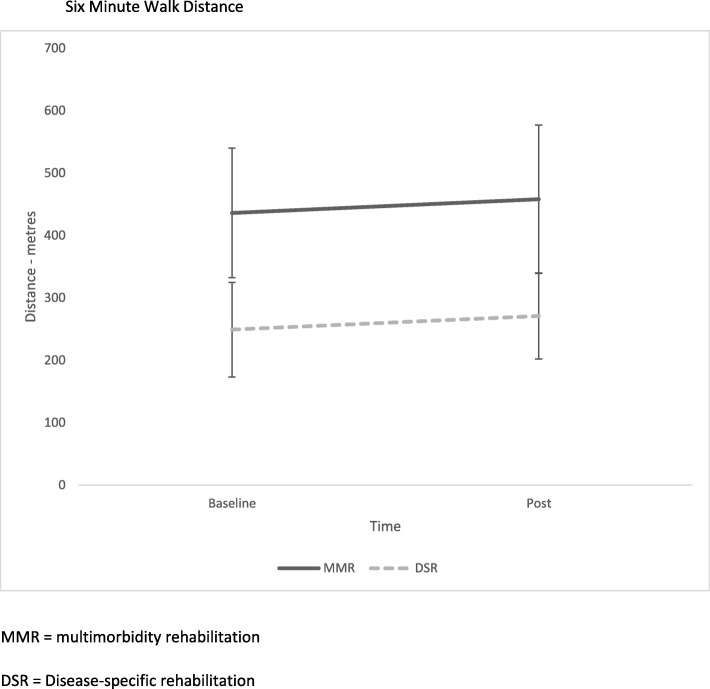
Six-minute walk distance

There was no significant difference in mean change in 6MWD from baseline to post-intervention between the groups, with the MMRG achieving a mean improvement of 22 (45) m (95% CI − 16 to 60) and the DSRG achieved a mean improvement of 22 (57) m (95% CI − 69 to 114) (Fig. 2). The data displayed in Fig. 2 reflect the numbers analyzed for each group accounting for withdrawals and losses to follow up (detailed in Fig. 1). In both groups, 50% of participants achieved the minimal important difference (MID) of at least 30 m [38].

There were no significant differences between the groups for improvement in AQoL, Katz ADL index, and EQ-5D-5 L (Table 3). There was a mean increase in the AQoL utility score for both groups, with a greater mean increase in the DSRG compared to the MMRG. Two participants from each group achieved the MID of 0.06 in the AQoL [47].

**Table 3:** Patient-centered outcome measures

	Multimorbidity Rehabilitation Group (MMRG) (*n* = 8)				Disease-Specific Rehabilitation Group (DSRG) (*n* = 5)				Difference between groups	
	Baseline	Post	Change	95% CI	Baseline	Post	Change	95% CI	Mean	95% CI
Six-min walk distance (m), mean (SD)	436	458	22 (45)	− 16–60	249 (*n* = 4)	271 (*n* = 4)	22 (57)	− 69–114	0	− 67–67
AQoL utility, mean (SD)	0.684 (0.204)	0.709 (0.222)	0.025 (0.136)	− 0.089–0.139	0.566 (0.271)	0.620 (0.257)	0.054 (0.363)	− 0.397–0.505	− 0.029	− 0.336–0.278
EQ-5D-5 L visual analogue scale, mean (SD)	73 (10)	76 (19)	3 (18)	− 12–18	55 (17) (*n* = 4)	53 (5) (*n* = 4)	− 3 (13) (*n* = 4)	− 24–19	6	− 17–28
St George Respiratory Questionnaire, mean (SD)	49 (16) (*n* = 3)	37 (20) (*n* = 3)	− 12 (19) (*n* = 3)	− 60–36	48 (*n* = 1)	49 (*n* = 1)	1 (*n* = 1)	N/A	− 12	− 109–84
Minnesota Living with Heart Failure Questionnaire	22 (*n* = 1)	13 (*n* = 1)	− 9 (*n* = 1)	N/A	N/A	N/A	N/A	N/A	N/A	N/A
Katz ADL index, mean (SD)	5.38 (1.79)	5.88 (0.34)	0.5 (2)	− 1.05–2.05	4.8 (0.84)	4.8 (1.10)	0 (2)	− 2.15–2.15	0.50	− 1.77–2.77

95% confidence interval represents comparison between groups for change over the course of the program

*n* number, *CI* confidence interval. *AQoL* assessment of quality of life, *SD* standard deviation, *ADL* activities of daily living, *EQ-5D-5 L* EuroQol-5D-5 L, *N/A* not applicable

Nine of the participants returned their daily diaries, with resource utilization recorded (MMRG *n* = 5; DSRG *n* = 4). All the participants had GP visits during the trial. The total number of GP visits were similar between groups (MMRG 10 vs DSRG 11). There was no significant difference in the mean number of GP visits between groups (MMRG 2 (1) vs DSRG 3 (2)). No participants had ED presentations, but two participants were admitted to the hospital, one from each group. Due to an error during the trial period, an outcome measure reported in the trial registry (Short Form 36 (SF-36)) was not collected, with the SF-36 form not included in the outcome measure packs during data collection.

## Discussion

This pilot study showed that it was feasible to conduct multimorbidity rehabilitation programs in people with multiple chronic diseases. The lack of adverse events during the multimorbidity program suggests this model was safe to conduct. This pilot trial supports the performance of a larger RCT in regard to recruitment, enrolment, consent, randomization, and undertaking of multimorbidity rehabilitation exercise and education sessions.

The similar number of sessions attended in both groups shows that people were willing to attend an outpatient exercise rehabilitation program that focused on multimorbidity compared to disease-specific groups. The prescribed exercise program was completed by participants when delivered by physiotherapy and nursing staff concurrently supervising people with different disease types. The completion rates for both groups were similar or better than those reported for disease-specific programs worldwide. In the UK, completion rates have been reported at 7% of those enrolled in pulmonary rehabilitation [48] and completion rates of cardiac rehabilitation in South Australia were 13% [49].

The procedures required to conduct a RCT were feasible in this trial. The well-established disease-specific rehabilitation programs of cardiac, HF, and pulmonary rehabilitation at the health network contributed to the ability to fully recruit. Only 61 people were required to be screened to fully recruit to this trial, which was 3.6 times the number required for enrollment. This should translate into achievable recruitment targets for a larger RCT. It was anecdotally reported by assessors and some participants that assessments were time-consuming due to questionnaire burden and outcome measures used. To make a larger trial less cumbersome and costly, refining the number of questionnaires and outcome measures would be beneficial. The reasons for non-enrollment in the trial were similar to the anecdotal barriers experienced for people attending existing disease-specific services at the health network, including unstable or uncontrolled disease, poor exercise capacity (an inability to walk 50 m), not interested in attending an exercise program, not wanting physiotherapy, and work commitments.

The education sessions for the MMRG were a novel part of this trial, with the objective of enhancing skills in disease self-management. It has been reported that education programs using self-management skill training are more effective in improving clinical outcomes than information-only education [50]. In future research, potential options for measuring the impact of the difference in the education sessions may include identifying a suitable tool for measuring education. The new model of multimorbidity rehabilitation had the novel aspect of participants attending a rehabilitation program with other people who had different diseases compared to them. There is potential value in investigating participants’ perceptions of peer support within rehabilitation and whether this influences satisfaction, attendance, and completion rates. Presently, an investigation into peer support largely focuses on programs delivered by trained individuals and not that achieved by informal support between peers within a rehabilitation group. For these novel aspects of multimorbidity rehabilitation, using focus groups with the participants and educators to gather qualitative data could provide meaningful information. This was not performed in this trial due to resources, but would be very valuable data to collect to inform the optimal design of a rehabilitation intervention for any future large trial.

Multimorbidity measures were used in this trial to describe a complex population. There is no gold standard measure of multimorbidity, and the tools available examine differing aspects of disease and burden. The FCI and MULTIPleS appeared to be the most suitable for a larger scale trial in terms of population suitability, ease of use, and information obtained. The FCI is simple to administer and score, with yes/no responses and a total of single scores [32]. This is an appropriate measure for use in a trial researching exercise capacity and rehabilitation as it was designed to focus on physical function [32]. The MULTIPleS measures a participant’s illness perception, which can affect people’s self-management of diseases and enable them to make sense of their conditions [33]. Physical function and disease self-management are important aspects of exercise rehabilitation, and therefore, the FCI and MULTIPleS are valuable measures. As previously stated, the DUSOI was a difficult measure to use with several issues encountered. The CIRS(G) was a time-consuming measure to administer. It was also difficult to obtain all required information to accurately score each category, with participants not undergoing investigations or results not being available. The clinical expertise of blinded assessors can affect the accurate scoring of the CIRS(G) due to the decision process required to clarify complex medical problems or their severity [51]. For a future larger scale trial, these difficulties could be avoided by carefully selecting the most relevant and applicable outcome measures.

This trial was limited in the estimate of potential intervention effect due to the small sample size, as expected in a feasibility study. This sample size was chosen to accommodate the number of participants who could attend the MMRG with available resources, an acceptable approach in pilot trials [46]. A larger RCT is needed to draw conclusions about the impact of a multimorbidity rehabilitation program on clinical outcomes. However, the ability of multimorbidity rehabilitation to accommodate people with different disease types and allow people to attend programs that run at different times throughout a week may create a more flexible model which may positively influence reported barriers of program timing and disruption to usual routines [52]. This is important with current pressure on healthcare resources and the growing burden of chronic diseases.

The results of this study allow estimation of sample sizes for a future randomized controlled trial comparing multimorbidity and disease-specific rehabilitation. To detect a clinically meaningful improvement for the primary outcome measure of 6MWT with 80% power, 114 participants are required [53]. This assumes a clinically meaningful difference between groups of 30 m, based on the well-established minimal important difference for 6MWT derived in patients with chronic respiratory disease [38], and assumes a SD of change in 6MWD of 57 m, based on data collected in this trial for the DSRG. To detect a clinically meaningful improvement for the secondary outcome of EQ-5D-VAS with 80% power, 214 participants are required [53]. This assumes a difference between groups of 6.9 points, which is the MID for the EQ-5D-VAS derived in the COPD population [54], and a SD of change in EQ-5D of 18, based on data from Table 3. Given the large confidence intervals, these estimations for adequate power should be interpreted with caution. Through the high prevalence of multimorbidity, these sample sizes should be readily achieved.

## Conclusions

It was feasible to conduct multimorbidity rehabilitation programs in people with chronic diseases. This provides a sound basis upon which to conduct a larger RCT comparing disease-specific and multimorbidity rehabilitation exercise and education sessions, from which definitive conclusions regarding efficacy can be made. This may assist in the development of effective healthcare models in the multimorbidity population.

## Additional file

Additional file 1: Supplemantary material. (DOC 103 kb)

## Abbreviations

6MWD: 6-min walk distance
6MWT: 6-min walk test
ADL: Activities of daily living
AQoL-4D: Assessment of Quality of Life
CI: Confidence interval
CIRS(G): Cumulative Illness Rating Scale for Geriatrics
COPD: Chronic obstructive pulmonary disease
DSRG: Disease-specific rehabilitation group
DUSOI: Duke Severity of Illness Checklist
ED: Emergency department
EQ-5D: EuroQol-5D-5 L
FCI: Functional Comorbidity Index
GP: General practitioner
HF: Heart failure
HRQoL: Health-related quality of life
IQR: Interquartile range
Katz ADL index: Katz Index of Independence in Activities of Daily Living
MID: Minimal important difference
MLHF: Minnesota Living with Heart Failure Questionnaire
MMRG: Multimorbidity Rehabilitation Group
MULTIPleS: Multimorbidity Illness Perception Scale
RCT: Randomized controlled trial
SD: Standard deviation
SF-36: Short form 36
SGRQ: St. George’s Respiratory Questionnaire
SPSS: Statistical Package for the Social Sciences
UK: United Kingdom
