# Supplementary material for: Multimorbidity rehabilitation versus disease-specific rehabilitation in people with chronic diseases: a pilot randomized controlled trial
Source: Pilot Feasibility Stud. 2018 Nov 29;4:181. doi: 10.1186/s40814-018-0369-2 (PMC6267787; doi:10.1186/s40814-018-0369-2)
Supplement: Supplementary file 1 — Supplemantary material. (DOC 103 kb) [file 40814_2018_369_MOESM1_ESM.doc]

**Online Supplement**

**Methods**

*Multimorbidity diagnosis*

A detailed list of chronic conditions that meet the definition of multimorbidity are outlined in Table 1 [1].

| Table 1. List of conditions for definition of multimorbidity | | | |
| --- | --- | --- | --- |
| **Condition** | **Diagnosis** | **Condition** | **Diagnosis** |
| Hypertension | Ever recorded | Alcohol problems | Ever recorded |
| Depression | Recorded in past 12 months OR ≥ 4 anti-depressant prescriptions (excluding low-dose tricyclics) in last 12 months | Other psychoactive substance misuse | Ever recorded |
| Painful condition | ≥ 4 prescription only medicine analgesic prescriptions in last 12 months OR ≥ 4 specified anti-epileptics in the absence of epilepsy | Treated constipation | ≥ 4 laxative prescriptions in last year |
| Asthma | Ever recorded and any prescription in the past 12 months | Stroke and transient ischemic attack | Ever recorded |
| Coronary heart disease | Ever recorded | Chronic kidney disease | Ever recorded |
| Treated dyspepsia | ≥ 4 prescriptions in the last 12 months BNF 0103% excluding antacids AND NOT ≥ 4 NSAIDS or ≥ 4 aspirin/clopidogrel | Diverticular disease of intestine | Ever recorded |
| Diabetes | Ever recorded | Atrial fibrillation | Ever recorded |
| Thyroid disorders | Ever recorded | Peripheral vascular disease | Ever recorded |
| Rheumatoid arthritis, other inflammatory polyarthropathies and systematic connective tissue disorders | Ever recorded | Heart failure | Ever recorded |
| Hearing loss | Ever recorded | Prostate disorders | Ever recorded |
| Chronic obstructive pulmonary disease | Ever recorded | Glaucoma | Ever recorded |
| Anxiety, neurotic, stress related and somatoform disorders | Recorded in past 12 months OR ≥ 4 anxiolytic/hypnotic prescriptions in last 12 months OR ≥ 4 10/25mg amitriptyline in last 12 months and don’t meet Pain criteria | Epilepsy (currently treated) | Ever recorded AND antiepileptic prescription in last 12 months |
| Irritable bowel syndrome | Ever recorded OR ≥ 4 prescription only antispasmodic prescription in past 12 months | Dementia | Ever recorded |
| New diagnosis of cancer in past 5 years | Recorded in past 5 years | Schizophrenia (and related psychosis) or bipolar disorder | Ever recorded/in last 12 months OR lithium prescribed in last 168 days |
| Psoriasis or eczema | Ever recorded AND ≥ 4 prescriptions in last 12 months | Migraine | ≥ 4 prescription only medicine anti-migraine prescriptions in last year |
| Inflammatory bowel disease | Ever recorded | Blindness/low vision | Ever recorded |
| Chronic sinusitis | Ever recorded | Anorexia or bulimia | Ever recorded |
| Learning disability | Ever recorded | Bronchiectasis | Ever recorded |
| Parkinson’s disease | Ever recorded | Viral hepatitis | Ever recorded |
| Multiple sclerosis | Ever recorded | Chronic liver disease | Ever recorded |

*Full exclusion criteria:*

- an inability to walk greater than fifty meters;
- severe cognitive impairment;
- psychiatric or intellectual disability which would limit the ability to participate in an exercise class with distant supervision or the ability to complete outcome measures (defined as mini-mental state exam (MMSE) <= 18 points) [2];
- pulmonary hypertension with recent history of dizziness or syncope on exertion (must have medical clearance if mean pulmonary artery pressure > 50 mm Hg);
- acute pulmonary embolus;
- interstitial lung disease;
- unstable cardiovascular disease (e.g. unstable angina, uncontrolled arrhythmia, New York Heart (NYH) Class 4 chronic heart failure (CHF), uncontrolled hypertension, diastolic pressure > 95 mm Hg);
- absolute contraindications to exercise (e.g. severe orthopedic/neurological deficit; severe uncontrolled pain: surgical or medical (including active transmissible infectious disease) restrictions to mobilization/rehabilitation (e.g. diabetic foot);
- severe ischemic vascular disease;
- advanced neuropathy/retinopathy which would compromise the ability to safely exercise;
- people already participating in a structured exercise rehabilitation program from a community or external provider;
- uncontrolled diabetes;
- uncontrolled epilepsy or seizures;
- extensive brain, skeletal or visceral metastases, life expectancy considered to be less than 12 months;
- known thrombocytopenia (<50×109/l) or severe neutropenia (neutropenia defined as absolute neutrophil count < 500/µL; profound neutropenia defined as ANL < 100 neutrophils/mm^3^ [2];
- room air desaturation at rest < 85%;
- abnormal and untreated moderate anemia (80-109 g/L);
- pregnant women.

*Exercise details*

*Aerobic component*: Comprising of walking (corridor or treadmill) and stationary cycling; a total of 30 minutes, for 15 minutes each. The initial walking prescription was calculated at 80% of peak walking speed or distance [3, 4] and stationary cycling intensity was calculated at 60-80% of maximum work rate estimated from the 6-minute walk test (6MWT) [5]. Exercise prescription was progressed using a rating of perceived exertion (RPE) Borg scale (6-20) and dyspnea modified Borg scale, aiming for a RPE score of 12-14 and a dyspnea score of 3-4, correlating to moderate intensity exercise [6].

*Resistance component:* Upper and lower limb exercises using free weights with four upper limb and three lower limb exercises. The initial load corresponded to 10-12 repetition maximum (RM). A 10-12 RM is the weight that can be lifted correctly and comfortably at least 10 times, but not more than 12 [7]. Progression was undertaken using a RPE Borg scale (6-20), aiming for a RPE score of 12-14. Usual cessation/withdrawal and safety criteria for chronic disease rehabilitation applied to all participants, which included a change in a participant’s medical condition that deemed them unsuitable for exercise (for further detail, see section below). Supplemental oxygen was delivered if SpO_2_ was <88% during exercise on room air and was titrated to maintain a SpO_2_ > 90%.

Usual cessation/withdrawal criteria for chronic disease rehabilitation applied to all participants as follows:

- a change in a participant’s medical condition that made them unsuitable for exercise;
- non-attendance (if patient fails to attend six consecutive sessions);
- a participant withdrawal from the program.

For the safety criteria, exercise was ceased if a participant displayed the following observations during group exercise:

- heart rate (HR) > 160bpm;
- blood pressure (BP) > 180 mmHg or <90 mmHg (systolic) and > 110 mmHg or < 60 mmHg (diastolic);
- SpO_2_ < 88% (exercise temporarily ceased and resumed when SpO2 reached 88% or supplemental oxygen supplied);
- diaphoretic, pale or dizzy;
- room air desaturation at rest < 85%;
- syncope, dizziness, onset of angina or chest pain;

Exercise was not commenced if the participants displayed the following observations:

- fever > 38.0,
- new known thrombocytopenia (<50×109/l) or severe neutropenia (neutropenia defined as absolute neutrophil count < 500/µL; profound neutropenia defined as ANL < 100 neutrophils/mm^3^ [8].

*Outcome measures*

Measures of multimorbidity included:

The Cumulative Illness Rating Scale for Geriatrics (CIRS(G)) [9], which consists of 14 body system categories, providing a severity scale for each domain [10]. The CIRS(G) has been successfully applied in medically impaired elderly subjects with good interrater reliability and face validity [9]. The Functional Comorbidity Index (FCI) [11] is an 18-item index based on diagnosis of comorbid diseases such as arthritis, COPD, depression and diabetes. The index is scored with one point per disease with the scores summed to provide a single total score; the higher the score, the greater number of co-morbidities in an individual. The Multimorbidity Illness Perception Scale (MULTIPleS) [12] examines five domains of emotional representations, treatment burden, prioritizing conditions, causal links and activity limitations. This measures the impact of multimorbidity on illness perceptions, adjustments, clinical outcomes, quality of life and costs in this population, with demonstrated validity and reliability. The Duke Severity of Illness Checklist (DUSOI) [13] measures the severity of a person’s illness. It comprises of four parameters for each diagnosis, including symptoms, complications, prognosis without treatment and treatment potential.

**Results**

*Other reasons for declining to participate in the trial (n = 1 each)*:

- time issues
- not wanting any physiotherapy
- not wanting to do exercise
- being main carer for a spouse
- participating in another trial
- work commitments
- living in another state

*Overall prevalence of comorbidities by group*

| Table 2. Prevalence of comorbidities | | |
| --- | --- | --- |
| **Other Comorbidity** (n, %) | **Multimorbidity**  **(n = 9)** | **Disease Specific**  **(n = 7)** |
| Anxiety | 2 (22%) | 0 (0%) |
| Asthma | 2 (22%) | 1 (14%) |
| Atrial Fibrillation | 1 (11%) | 1 (14%) |
| Chronic Kidney Disease | 0 (0%) | 1 (14%) |
| Chronic Liver Disease | 1 (11%) | 0 (0%) |
| Chronic Obstructive Pulmonary Disease | 3 (33%) | 2 (29%) |
| Coronary Heart Disease | 8 (89%) | 4 (57%) |
| Depression | 1 (11%) | 3 (43%) |
| Diabetes | 4 (44%) | 4 (57%) |
| Diverticular Disease of Intestines | 0 (0%) | 1 (14%) |
| Glaucoma | 1 (11%) | 0 (0%) |
| Heart Failure | 1 (11%) | 2 (29%) |
| Hypertension | 8 (89%) | 5 (71%) |
| Rheumatoid Arthritis | 0 (0%) | 2 (29%) |
| Schizophrenia/Bipolar Disorder | 0 (0%) | 1 (14%) |
| Stroke/Transient Ischaemic Attack | 1 (11%) | 0 (0%) |
| Thyroid Disorders | 0 (0%) | 1 (14%) |
| Treated Dyspepsia | 0 (0%) | 3 (43%) |

**Supplement References**
